# Approximate Bayesian computation supports a high incidence of chromosomal mosaicism in blastocyst-stage human embryos

**DOI:** 10.1101/2024.11.26.625484

**Published:** 2024-12-02

**Authors:** Qingya Yang, Sara A. Carioscia, Matthew Isada, Rajiv C. McCoy

**Affiliations:** 1 Department of Biology, Johns Hopkins University, Baltimore, MD, USA 21218

## Abstract

Chromosome mis-segregation is common in human meiosis and mitosis, and the resulting aneuploidies are the leading cause of pregnancy loss. Preimplantation genetic testing for aneuploidy (PGT-A) seeks to prioritize chromosomally normal embryos for transfer based on genetic analysis of a biopsy of approximately five trophectoderm cells from blastocyst-stage *in vitro* fertilized (IVF) embryos. While modern PGT-A platforms classify these biopsies as aneuploid, euploid, or mosaic (possessing a mixture of normal and aneuploid cells), the underlying incidences of aneuploid, euploid, and mosaic embryos and the rates of meiotic and mitotic error that produced them remain largely unknown. To address this knowledge gap, we paired a recent method for embryo simulation with approximate Bayesian computation (ABC) to infer rates of meiotic and mitotic error that best explain published PGT-A data. By simulating from these posterior distributions, we also evaluated the chromosomal status of entire embryos. For a published clinical sample, we estimated a 39–43% probability of meiotic error per meiosis, as well as a 1.0–3.0% probability of mitotic error per mitosis, depending on assumptions about spatial clustering of aneuploid cells within mosaic embryos. In addition, our analyses suggest that less than 1% of blastocysts are fully euploid, and that many embryos possess low-level mosaic clones that are not captured during biopsy. These broad conclusions were relatively insensitive to potential misclassification of mosaic biopsies. Together, our work helps overcome the limitations of embryo biopsies to estimate the fundamental rates of cell division errors that are the main causes of human pregnancy loss.

## Introduction

It is estimated that only approximately half of human conceptions survive to birth. The leading cause of human pregnancy losses—most of which occur very early in development—is abnormality in chromosome number (aneuploidy).^1–3^ Chromosomes frequently mis-segregate during (predominantly maternal) meiosis, producing embryos with homogeneous forms of aneuploidy that affect all cells.^4,5^ However, errors may also arise during postzygotic cell divisions, producing “mosaic” embryos with two or more karyotypically distinct cell lineages.^6^ The first two cell divisions appear especially susceptible to such mitotic errors.^7^

While many forms of aneuploidy are lethal during cleavage-stage development, other forms of aneuploidy, especially in mosaic form, can survive to the blastocyst stage and beyond.^8–10^ Considering the potential fitness consequences of aneuploidy, preimplantation genetic testing for aneuploidy (PGT-A) seeks to improve IVF outcomes by prioritizing chromosomally normal (i.e., euploid) embryos for transfer based on genetic analysis of embryo biopsies,^11^ though its clinical efficacy is the subject of long-standing debate.^12–17^ Current implementations of PGT-A involve sequencing or single nucleotide polymorphism (SNP) microarray-based genotyping of DNA extracted from trophectoderm biopsies of blastocyst-stage embryos, 5–6 days after fertilization.^18^ These modern, sensitive PGT-A platforms have revealed evidence of mosaic aneuploidy within 2–13% of embryo biopsies, posing a dilemma for clinical diagnosis and management.^19^

One notable limitation of PGT-A is its dependence on a single, spatially restricted biopsy of 5–10 trophectoderm cells, which may or may not represent the constitution of the remaining >100 cells of the blastocyst-stage embryo, including the inner cell mass.^20,21^ While studies employing multiple biopsies of individual embryos^22,23^ or single-cell genomic analysis^24,25^ have offered additional insight, they are typically limited in sample size of embryos and/or availability of ground-truth data for comparison. Thus, the clinical literature is replete with reports of the relative incidences of euploid, mosaic, and aneuploid embryo biopsies as diagnosed by PGT-A, but the underlying incidence of mosaic embryos and the fundamental rates of meiotic and mitotic errors that produced them remain largely obscure.

Approximate Bayesian computation (ABC) offers a statistical framework for estimating unknown parameters given summary statistics from observed data and a generative model for simulating data under a specified set of parameters.^26^ In addition to point estimates of the parameters, the posterior distributions produced by ABC provide measurements of uncertainty that can be summarized, for example, with credible intervals (CIs). ABC has found growing applications in the fields of population genetics, ecology, and epidemiology, in concert with the development of powerful computational simulations.^27^

Skinner et al.^28^ recently developed an R package, Tessera, to model the growth and distribution of aneuploid cells in a three-dimensional early embryo, followed by a trophectoderm biopsy to mimic PGT-A. In our study, we used Tessera to simulate embryos over a range of meiotic and mitotic error rates. We then applied ABC to identify the subset of embryos whose biopsy results best reflect published PGT-A data from IVF clinics, in turn obtaining posterior probability estimates of meiotic and mitotic error rates and incidences of euploid, mosaic, and aneuploid embryos. Together, our study helps overcome the limited focus on embryo biopsies to reveal knowledge of fundamental parameters shaping the chromosomal landscape of human preimplantation embryos.

## Results

### ABC enables inference of rates of meiotic and mitotic error

**Figure 1:**
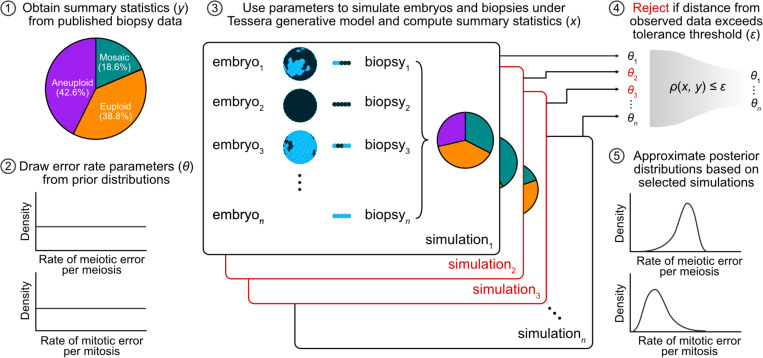
Schematic of the ABC approach for inferring meiotic and mitotic error rates. For each simulation trial, probabilities of meiotic and mitotic error (*θ*) are each randomly drawn from a uniform distribution ranging from 0 to 1. These values are used to determine the number of cells that will be aneuploid (represented as dark blue in the schematic) versus normal disomic (light blue) in the final embryo. This number is converted to a proportion and then used as input to the Tessera package. For each combination of error parameters (1,000 chosen) and each of three fixed values for dispersal (0, 0.5, 1), 1,000 embryos are generated and biopsied. For each embryo, 8 mitotic divisions are simulated (producing a total of 256 cells), and one 5-cell biopsy is obtained. Using the adaptive method described by Lenormand et al.,^29^ ABC selects the set of simulations that produce summary statistics (proportions of euploid, mosaic, and aneuploid biopsies) that best reflect the observed biopsy data.^10^ The error rates that produced the selected simulations are used to approximate the posterior distributions of these parameters.

We used Tessera to simulate large samples of human embryos and biopsies within the ABC framework (see Methods; Figure 1). Specifically, given a rate of meiotic error per meiosis (i.e., the probability of producing an aneuploid zygote, ignoring the distinction between maternal and paternal meiosis) drawn from a uniform prior distribution, a rate of mitotic error per mitosis (i.e., the probability of a postzygotic cell division producing aneuploid daughter cells) drawn from a uniform prior distribution, and a rate of dispersal (i.e., the randomness of spatial organization of aneuploid cells within mosaic embryos) fixed at 0, 0.5, or 1, we simulated trials of 1,000 embryos each. We selected these dispersal values to span the entire parameter range, as the spatial distribution of aneuploid cells within mosaic embryos remains largely unknown (see Discussion). We then simulated biopsies of five spatially adjacent cells from each embryo, and summarized each trial based on the proportion of euploid, mosaic, and aneuploid biopsies. Within the ABC framework, we compared these proportions to those from published PGT-A results from 73,218 embryos across 5 clinics (38.8% euploid, 18.6% mosaic, and 42.6% aneuploid)^10^ and rejected simulations whose distance from the published data exceeded a given tolerance threshold. For the remaining selected simulations, we used the corresponding values of the meiotic and mitotic error rate parameters to approximate posterior probability distributions. The sequence of tolerance levels, stopping criteria, and bias correction were obtained using the adaptive method described by Lenormand et al.^29^ and implemented with the EasyABC package in R.^30^

**Figure 2:**
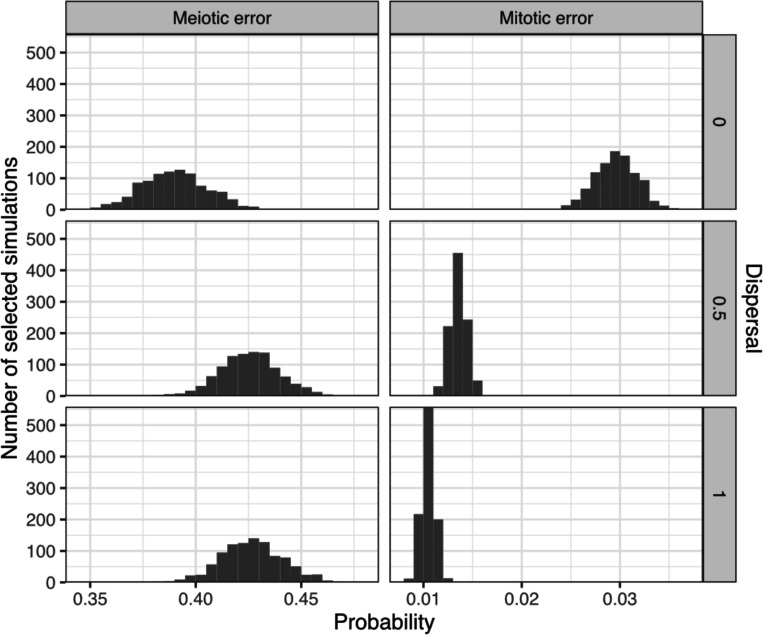
Posterior probabilities of meiotic and mitotic error. Posterior distributions of meiotic and mitotic error probability (per meiosis and mitosis, respectively) based on 3,000 simulations (1,000 simulations per level of dispersal) selected by ABC as best matching published data.

At a dispersal level of 0, the posterior mean estimates of the meiotic and mitotic error rates were 39% (95% CI [36%, 42%]) and 3.0% (95% CI [2.5%, 3.4%]), respectively; at a dispersal level of 0.5, the posterior mean estimates of the meiotic and mitotic error rates were 43% (95% CI [40%, 45%]) and 1.4% (95% CI [1.2%, 1.5%]), respectively; and at a dispersal level of 1, the posterior mean estimates of the meiotic and mitotic error rates were 43% (95% CI [40%, 46%]) and 1.0% (95% CI [0.91%, 1.2%]), respectively. These estimates are summarized in Figure 2 and Table S1 and indicate that higher dispersal levels (i.e., more random spatial organization of aneuploid cells) require lower estimates of mitotic aneuploidy rates and modestly higher estimates of meiotic aneuploidy rates to explain the observed data. This result is intuitive, as higher dispersal levels make it more likely to detect mosaic aneuploidy within a given biopsy, such that relatively lower mitotic error rates would be required to explain the observed data.

**Figure 3:**
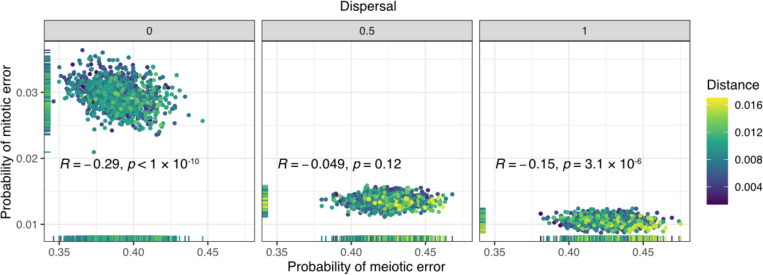
Relationship between inferred meiotic and mitotic error rates. Meiotic versus mitotic error rate, stratified by dispersal level, across 3,000 simulations selected by ABC. The coefficient (*R*) and p-values of Pearson correlations between inferred meiotic and mitotic error rates are displayed in each panel.

For the selected simulations, we observed a weak negative correlation between the inferred meiotic and mitotic error rates at each dispersal level (Figure 3). The correlation was relatively more pronounced when the dispersal level was low, reflecting the fact that spatial clustering of cells makes it more likely that a mitotic aneuploidy could mimic a meiotic aneuploidy (or euploid embryo) by affecting all cells (or no cells) within a biopsy. Higher assumed rates of dispersal make these forms of aneuploidy more distinguishable in biopsy data, reducing the correlation.

### Evidence that few embryos are fully euploid

We next used the ABC results to examine the set of simulated embryos that produce the biopsy results that best match Viotti et al.^10^ (Table S2). To this end, for each dispersal level, we generated embryos from the posterior distributions of meiotic and mitotic error rates and examined their entire cellular content. In contrast to the proportions of euploid, aneuploid, and mosaic biopsies, this allowed us to quantify the underlying proportions of the fully euploid, fully aneuploid, and mosaic embryos (and their corresponding proportions of aneuploid cells) that produced these data—quantities that remain largely unknown within the field.

**Figure 4:**
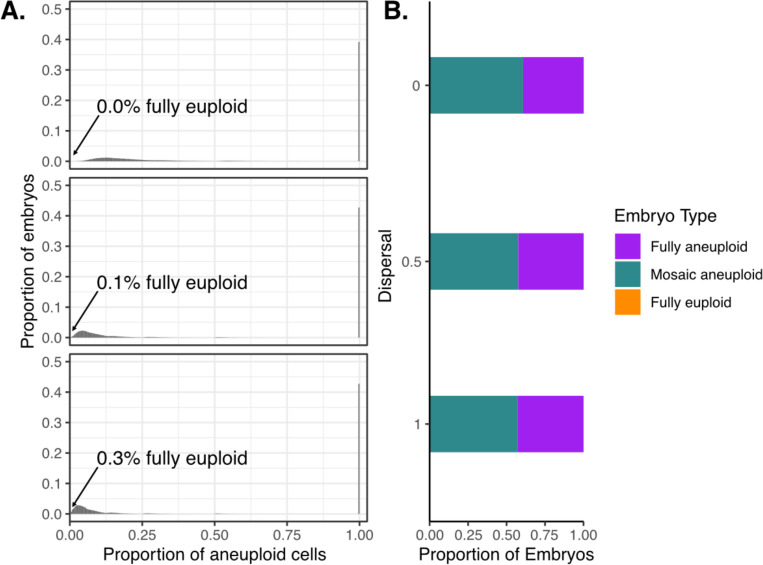
Posterior predictive samples of embryos that are fully aneuploid, mosaic aneuploid, and fully euploid. **(A)** Distribution of proportions of aneuploid cells in embryos simulated based on meiotic and mitotic error rates drawn from the posterior distributions (10,000,000 embryos for each level of dispersal; Figure 2). The arrow marks the percentage of fully euploid embryos (0 aneuploid cells) in the posterior predictive sample for each dispersal level (0.0%, 0.1%, and 0.3%). **(B)** Proportions of fully aneuploid, mosaic aneuploid, and fully euploid embryos in the posterior predictive sample, stratified by dispersal level.

Strikingly, we found that regardless of assumptions about dispersal, fewer than 1% of embryos from the posterior predictive samples were entirely euploid (Figure 4; Table S2). Specifically, for simulated dispersal levels of 0, 0.5, and 1, we inferred that 0.0%, 0.1%, and 0.3% of embryos were fully euploid, respectively. A large proportion of embryos in selected simulations exhibited mosaicism, especially low-level mosaicism affecting <25% of cells. Higher levels of dispersal were consistent with lower levels of aneuploidy within mosaic embryos and higher proportions of aneuploid embryos (Figure 4). Intuitively, due to the small number of cells in biopsies, aneuploid cells of low-level mosaic embryos are rarely sampled and their embryo biopsies are classified as euploid. Within a mosaic embryo, higher levels of dispersal increase sensitivity for capturing one or more aneuploid cells within a biopsy. This effect is also apparent from the skewness of the distribution of the proportion of aneuploid cells within mosaic embryos, where higher simulated levels of dispersal require smaller proportions of aneuploid cells within mosaic embryos to explain the observed data (Figure 4).

### Inferences are robust to moderate rates of misclassification

**Figure 5:**
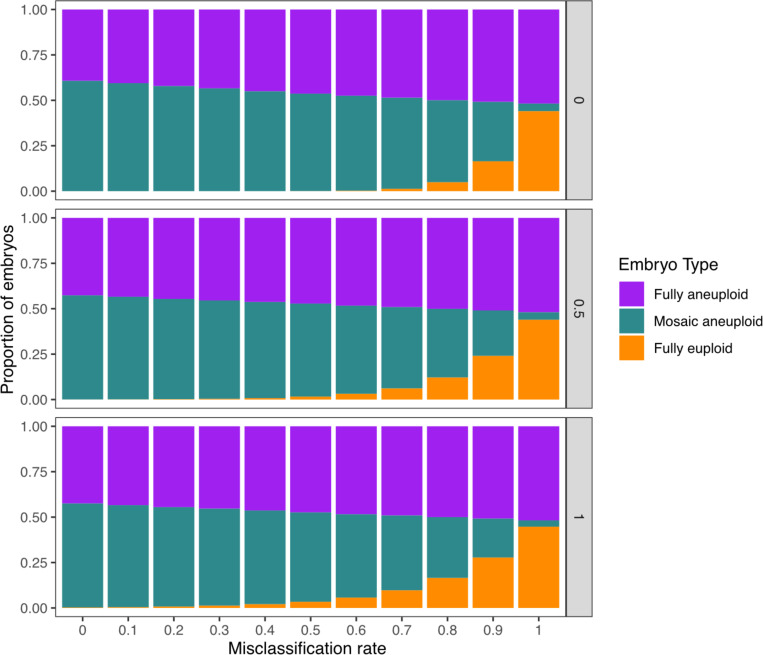
Inferred proportions of embryo types assuming varying levels of biopsy misclassification. 1,000 pairs of error rates were sampled from the posterior distributions generated by ABC with adjusted expected values (based on the rate of mosaic biopsy misclassification). Each error rate pair was used to generate 1,000 embryos, and the mean proportions of each biopsy type were plotted. The y-axis represents the proportion of false-positive mosaic biopsies that are supposed to be euploid or aneuploid in the expected value. The higher the misclassification rate, the fewer true mosaic biopsies in the reference clinical data. The maximum standard deviation of all embryo type averages is 4%.

In various perspective articles, authors have argued that mosaic biopsies may be over-diagnosed, as technical variability in depth of coverage (e.g., due to amplification artifacts) may be falsely interpreted as evidence of mosaicism.^31,32^ To address the possibility of biopsy misclassification and assess its impact on our results, we adjusted the reference data from Viotti et al.^10^ by evenly re-assigning a varying proportion of mosaic biopsies as either euploid or aneuploid, and then applied the same ABC process to this adjusted reference data (Figure S1). We then sampled error rate pairs from each of the newly inferred posterior distributions and generated embryos for downstream analysis. While the inferred incidence of mosaic embryos quantitatively declined with higher rates of misclassification, qualitative conclusions about the incidence of mosaic embryos were relatively insensitive to the mis-diagnosis of embryo biopsies (Figure 5). Notably, even when assuming a mis-diagnosis rate as high as 70%, fewer than 10% of embryos were inferred as fully euploid, regardless of the assumed level of dispersal.

## Discussion

Aneuploidies are prevalent in human development and are the leading cause of pregnancy loss.^33^ Retrospective studies of PGT-A data typically report proportions of embryo biopsies classified as euploid, aneuploid, or mosaic. While potentially justified from a clinical perspective (as these are the classifications used to inform embryo prioritization), it is important to consider from a fundamental biological perspective that these results are based on a biopsy of ~5 spatially restricted trophectoderm cells from a blastocyst-stage embryo composed of >100 cells. The underlying incidences of fully euploid, fully aneuploid, and mosaic embryos remain largely unknown and contentious,^31,21,32^ and the fundamental rates of meiotic and mitotic chromosome mis-segregation that produce these patterns have not been estimated aside from live imaging studies of the first two postzygotic cell divisions.^7^ Here we used the statistical method ABC to address these limitations and achieve embryo-wide estimates of aneuploidy that best explain published clinical data.

Our most provocative conclusion is that the inferred proportion of mosaic embryos is much higher than the observed proportion of mosaic biopsies, and that very few embryos are fully euploid at the blastocyst-stage of development. This conclusion is consistent with studies performing multiple biopsies of individual embryos, which observed that “fully aneuploid” biopsies exhibited high concordance upon re-biopsy, suggesting their likely meiotic origins, whereas mosaic aneuploid biopsies exhibited low concordance, suggesting that they were relegated to a small number of cells.^23,22^ Recent studies leveraging single-cell sequencing data also support the conclusion that low-level mosaicism is widespread at the blastocyst stage, though were limited in sample size of embryos and ground-truth data for validation.^24,34,25,35^

As with all models, our results must be interpreted in light of several simplifying assumptions. One such assumption regards the spatial distribution of aneuploid cells within mosaic embryos, which remains largely unknown. Past work in mouse models of mosaicism reported an even distribution of aneuploid clones throughout post-implantation conceptuses (day 13.5 of embryonic development), with no significant enrichment in the placenta versus the fetus (though note that these mosaic aneuploidies were experimentally induced with reversine).^36^ To address this uncertainty, we performed simulations at varying dispersal levels ranging from even mixture (dispersal = 0) to complete spatial clustering (dispersal = 1) of aneuploid cells. Importantly, our major qualitative conclusions about the incidence of mosaic aneuploidy were relatively insensitive to assumptions about dispersal levels. Further research leveraging imaging and/or spatial genomic methods will be required to detail the spatial distribution of aneuploid cells within mosaic embryos and how these patterns may change throughout development.

For simplicity, our model represents the ploidy of a given cell as a binary variable (aneuploid vs. euploid) and does not consider the specific chromosome affected or the category of aneuploidy (gain vs. loss of maternal vs. paternal homologs). The main consequence of these simplifications is that we do not consider the possibility of “rescue” events where aneuploid lineages revert to euploidy due to subsequent mitotic errors. We believe that these simplifications are nevertheless justified to avoid model overparameterization, especially given current evidence that aneuploidy rescue is rare in blastocyst-stage human embryos (based on the paucity of uniparental disomy).^6^ Similarly, our model does not account for potential changes in rates of mitotic error across cell divisions, and indeed, the first two cell divisions are known to be particularly error-prone.^7^ The practical effect of this simplification is that we likely underestimate the rate of error for initial divisions, but overestimate the rate of error for later divisions. Allowing for such error rate change is an interesting future direction, but may again introduce too many additional parameters given the dimensionality of the target data.

Related to the above considerations, we emphasize that published PGT-A data are subject to several selection and ascertainment biases. One of the most important such biases is that only embryos that survive to the blastocyst stage and possess high morphological grades are typically biopsied and reported in retrospective clinical studies.^37,38,10^ Meanwhile, the approximately half of embryos that arrest prior to blastocyst formation are highly enriched for abnormal cell divisions and complex forms of chromosomal mosaicism, as well as meiotic aneuploidies to a lesser extent.^8,39–41^ As a consequence of these selection and ascertainment biases, our inferred rates of both meiotic and mitotic error are likely to be underestimates of the true rates across all embryos and should instead be interpreted as estimates for the clinically tested sample.

While meiotic aneuploidies are generally harmful for development, mosaic aneuploidies that persist to the blastocyst stage are potentially compatible with healthy birth.^42,43^ This hypothesis was recently supported by a non-selection clinical trial which demonstrated that embryos exhibiting mosaicism as assessed by PGT-A had pregnancy outcomes equivalent to euploid embryos,^9^ though this may depend on the specific definition of mosaicism.^44^ While numerous forms of pathogenic mosaic aneuploidy have been reported,^45^ current evidence suggests that mosaic aneuploidies identified in blastocyst-stage embryos are rarely detected at later stages of pregnancy or at birth^10^ (though see ^46^). This observation is consistent with a model of selection against aneuploid cells within mosaic embryos during peri- and post-implantation development, recently supported by data from mouse and human embryo models,^47,48,36^ as well as analyses of single-cell genomic data from human embryos.^47,24^ Future extensions of our model may consider such mechanisms of intra-embryo negative selection, especially if supplemented by data from additional embryonic and fetal time points.

Together, our work helps expand beyond the limited snapshot of embryo biopsies by estimating the fundamental rates of cell division errors that best explain clinical data. Understanding these basic error mechanisms, the evolutionary forces that shape them, and their consequences for development enhance basic understanding of human development, while building the foundations for future clinical innovations.

## Methods

For each sampled combination of meiotic and mitotic error rates, we simulated a single biopsy from each embryo using the Tessera R package.^28^ We then applied approximate Bayesian computation (ABC) using the R package EasyABC^30^ to infer posterior distributions of the meiotic and mitotic error rates by comparing the simulated biopsy data to published clinical data.^10^ Our approach is summarized in Figure 1.

### Embryo model abstraction

Meiotic errors were simulated as producing embryos with entirely aneuploid cells. Mitotic errors were simulated as a given division of a euploid cell producing two aneuploid daughter cells. Upon selecting a meiotic and mitotic error rate, we simulated 8 mitotic cell divisions for a total of 256 cells. We repeated each simulation trial across three fixed dispersal levels: 0 (complete clustering of aneuploid cells), 0.5 (intermediate clustering of aneuploid cells), and 1 (random distribution of aneuploid cells). For the purpose of abstraction, aneuploidy was defined as a binary variable, ignoring the identity of individual chromosomes (see Discussion).

### Biopsy data reference

The clinical data used as the target for ABC are derived from Viotti et al.^10^ and include 73,218 embryos biopsied from 5 different IVF centers between 2015 and 2020. Of these biopsies, 38.8% (28,431) were euploid, 18.6% (13,602) were mosaic, and 42.6% (31,185) were aneuploid. Embryos in that study were classified as euploid if, based on estimates from copy number analysis, fewer than 20% of the biopsied cells were aneuploid, as mosaic if 20–80% of the cells were aneuploid, and as aneuploid if more than 80% of the cells were aneuploid for any genomic region.^10^ This standard is described in detail in the Preimplantation Genetic Diagnosis International Society (PGDIS) statement, which is used as a reference to IVF procedures and decision-making regarding the transfer of mosaic embryos.^49^

### ABC algorithm

ABC involves simulating a large number of trials under a given model, comparing the simulation results to a set of target summary statistics, and identifying the parameters that best explain those data.^30^ Specifically, we used the adaptive population Monte Carlo ABC method from Lenormand et al.^29^ to automatically select a series of tolerance levels and stopping criteria. This method was demonstrated by the authors to produce high-quality estimates of the posterior distributions with fewer simulations.

We implemented this procedure with the ABC_sequential function and the methods=“Lenormand” argument in the EasyABC package in R. Meiotic and mitotic error rates were drawn from uniform distributions (ranging from 0 to 1, exclusive), and each selected error rate pair was used to simulate 1,000 embryos.^30^ The embryos and their biopsy summaries were then compared with the published clinical PGT-A results,^10^ and the posterior distributions were obtained.

### Posterior predictive distributions of embryos

To understand the characteristics of embryos given our inferences, we simulated 1,000,000 unique embryos by drawing meiotic and mitotic error rates from our posterior distributions (1,000 times), repeating this procedure for each of the three fixed dispersal levels. Proportions of fully euploid, fully aneuploid, and mosaic embryos were then tabulated for each dispersal level, as was the distribution of the proportion of aneuploid cells within mosaic embryos.

### Misclassification simulation

To investigate a scenario in which some biopsies classified as mosaic were in fact technical false positives, we re-classified mosaic biopsies from the Viotti et al.^10^ study as euploid and aneuploid (from 0% to 100%, incrementing in steps of 10%). Using these adjusted values as the new target, we repeated our ABC and posterior predictive sampling procedures described above across each of the three fixed dispersal levels.

## Supplementary Material

Supplement 1
