# Supplementary material for: Approximate Bayesian computation supports a high incidence of chromosomal mosaicism in blastocyst-stage human embryos
Source: bioRxiv. 2025 Jul 25:2024.11.26.625484. Originally published 2024 Dec 2. Preprint. [Version 2] doi: 10.1101/2024.11.26.625484 (PMC11642757; doi:10.1101/2024.11.26.625484)
Supplement: Supplement 1 [file media-1.pdf]

Supplementary Materials

	Dispersal = 0		Dispersal = 0.5		Dispersal = 1	
	Meiotic	Mitotic	Meiotic	Mitotic	Meiotic	Mitotic
**Mean**	0.40	0.063	0.57	0.021	0.58	0.015
**Pctl. 2.5**	0.34	0.054	0.54	0.018	0.55	0.014
**Pctl. 25**	0.38	0.059	0.56	0.020	0.57	0.015
**Pctl. 50**	0.40	0.062	0.57	0.020	0.58	0.015
**Pctl. 75**	0.42	0.065	0.58	0.021	0.59	0.016
**Pctl. 97.5**	0.45	0.071	0.60	0.023	0.60	0.017
**MAP**	0.40	0.063	0.57	0.020	0.58	0.015

Supplementary Table 1: Inferred rates of meiotic and mitotic error at varying levels of dispersal. The reported statistics are summaries of the corresponding posterior distributions. The 2.5 and 97.5 percentiles of the posterior distributions form the boundaries of the 95% credible intervals. The last row reports the maximum *a posteriori* (MAP) estimate (Capalbo et al. 2021).

	Dispersal = 0			Dispersal = 0.5			Dispersal = 1		
	**Euploid**	**Mosaic**	**Aneuploid**	**Euploid**	**Mosaic**	**Aneuploid**	**Euploid**	**Mosaic**	**Aneuploid**
**Mean**	0.23	0.19	0.58	0.23	0.19	0.58	0.23	0.19	0.58
**Std. Dev.**	0.0043	0.0031	0.0047	0.0032	0.0054	0.0056	0.0027	0.0048	0.0049
**Min.**	0.22	0.18	0.57	0.23	0.18	0.57	0.23	0.18	0.57
**Pctl. 25**	0.23	0.18	0.58	0.23	0.18	0.58	0.23	0.18	0.58
**Pctl. 50**	0.23	0.19	0.58	0.23	0.19	0.58	0.23	0.19	0.58
**Pctl. 75**	0.23	0.19	0.58	0.23	0.19	0.58	0.23	0.19	0.58
**Max.**	0.24	0.19	0.59	0.24	0.20	0.59	0.24	0.20	0.59

Supplementary Table 2: Biopsy results for the simulations selected by ABC. For all simulated levels of dispersal, the biopsy results closely matched the targets from the published data, supporting the model fit (Capalbo et al. 2021).

**Dispersal**	**Total aneuploid biopsies**	**Number of aneuploid biopsies originating from mosaic embryos**
0	581518	183313 (31.5%)
0.5	581303	7891 (1.4%)
1	583075	3975 (0.7%)

Supplementary Table 3: Proportion of aneuploid biopsies (i.e., biopsies where all 5 sampled cells were aneuploid) obtained from embryos that were actually mosaic. All embryos used for biopsy were from the posterior predictive samples generated from the distribution of meiotic and mitotic error rates selected at each dispersal level.

Embryo type	First biopsy	Second biopsy	Dispersal = 0	Dispersal = 0.5	Dispersal = 1
Mosaic aneuploid	Euploid	Euploid	108408 (47%)	150107 (64.9%)	156474 (68%)
Mosaic aneuploid	Euploid	Mosaic	67793 (29.4%)	80452 (34.8%)	73288 (31.9%)
Mosaic aneuploid	Euploid	Aneuploid	54450 (23.6%)	702 (0.3%)	49 (0%)
Mosaic aneuploid	Mosaic	Euploid	70813 (37.7%)	79995 (42.7%)	77195 (41.3%)
Mosaic aneuploid	Mosaic	Mosaic	59526 (31.7%)	102154 (54.5%)	106954 (57.2%)
Mosaic aneuploid	Mosaic	Aneuploid	57492 (30.6%)	5272 (2.8%)	2804 (1.5%)
Mosaic aneuploid	Aneuploid	Euploid	55148 (9.5%)	759 (0.1%)	72 (0%)
Mosaic aneuploid	Aneuploid	Mosaic	57261 (9.8%)	5410 (0.9%)	2891 (0.5%)
Mosaic aneuploid	Aneuploid	Aneuploid	70904 (12.2%)	1722 (0.3%)	1012 (0.2%)
Fully aneuploid	Aneuploid	Aneuploid	398205 (68.5%)	573412 (98.6%)	579100 (99.3%)
Fully euploid	Euploid	Euploid	NA	15 (0%)	161 (0.1%)

Supplementary Table 4: First and second biopsy summaries of embryos constructed from the posterior distributions of meiotic and mitotic error rates inferred with ABC. At each dispersal level, we also report the percentages of each combination of embryo type, first biopsy type, and second biopsy type out of all embryos of a given first biopsy type

Data set	Maternal Age
Capalbo et al. (2021)	Mean: 38.06<br>Standard deviation: 3.65
Clarke et al. (2023)	Median: 34.0<br>670 patients < 35<br>462 patients > 35
Munné et al. (2017)	Mean: 35.8
Rodrigo et al. (2020)	Mean: 38.6<br>Standard deviation: 6.2

Supplementary Table 5: Maternal ages of PGT-A patients across input datasets. Studies differed in reported measures of central tendency and variability.

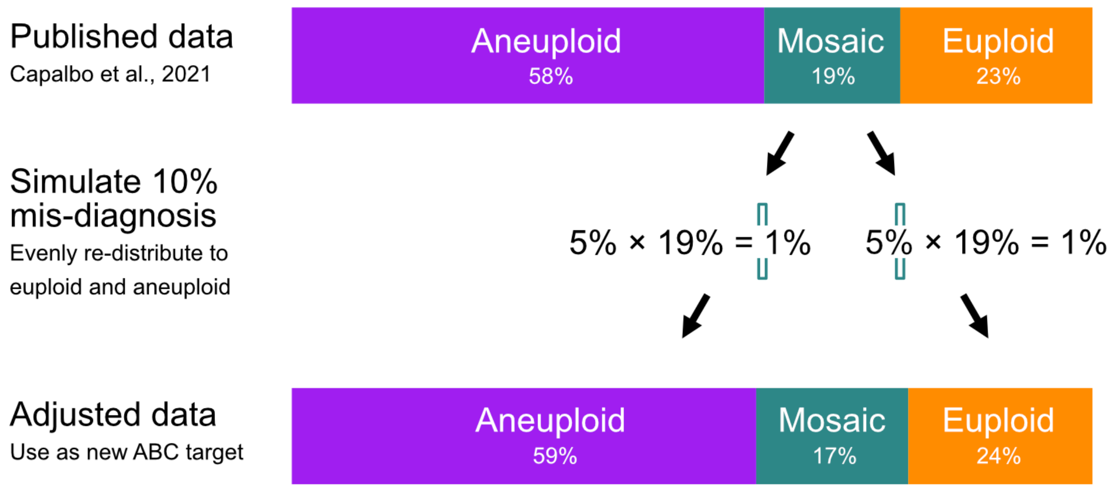

Supplementary Figure 1: Schematic of approach for simulating misclassification of mosaic embryos. We simulated varying levels of misclassification spanning from 0% to 100% in 10% increments. At each misclassification level, half of the mis-classified mosaic biopsies were assumed to be euploid and the other half aneuploid. These adjusted values were used as the new targets for ABC. The figure depicts an example of simulating a misclassification rate of 10% (Capalbo et al. 2021).
