# Supplementary material for: Approximate Bayesian computation supports a high incidence of chromosomal mosaicism in blastocyst-stage human embryos
Source: bioRxiv. 2024 Dec 2:2024.11.26.625484. Preprint. [Version 1] doi: 10.1101/2024.11.26.625484 (PMC11642757; doi:10.1101/2024.11.26.625484)
Supplement: Supplementary file 1 [file media-1.pdf]

Supplemental Tables

	Dispersal = 0		Dispersal = 0.5		Dispersal = 1	
	Meiotic	Mitotic	Meiotic	Mitotic	Meiotic	Mitotic
**Mean**	0.39	0.030	0.43	0.014	0.43	0.010
**Pctl. 2.5**	0.36	0.025	0.40	0.012	0.40	0.0091
**Pctl. 25**	0.38	0.028	0.42	0.013	0.42	0.010
**Pctl. 50**	0.39	0.030	0.43	0.014	0.43	0.011
**Pctl. 75**	0.40	0.031	0.43	0.014	0.44	0.011
**Pctl. 97.5**	0.42	0.034	0.45	0.015	0.46	0.012
**MAP**	0.39	0.029	0.43	0.014	0.43	0.011

Table S1: Inferred rates of meiotic and mitotic error at varying levels of dispersal. The reported statistics are summaries of the corresponding posterior distributions. The 2.5 and 97.5 percentiles of the posterior distributions form the boundaries of the 95% credible intervals. The last row reports the maximum *a posteriori* (MAP) estimate.

	Dispersal = 0			Dispersal = 0.5			Dispersal = 1		
	Euploid	Mosaic	Aneuploid	Euploid	Mosaic	Aneuploid	Euploid	Mosaic	Aneuploid
**Mean**	0.39	0.19	0.43	0.39	0.19	0.43	0.39	0.19	0.43
**Std. Dev.**	0.0039	0.0042	0.0050	0.0028	0.0055	0.0055	0.0028	0.0058	0.0058
**Min.**	0.38	0.18	0.42	0.38	0.17	0.41	0.38	0.17	0.41
**Pctl. 25**	0.39	0.18	0.42	0.39	0.18	0.42	0.39	0.18	0.42
**Pctl. 50**	0.39	0.19	0.43	0.39	0.18	0.43	0.39	0.19	0.43
**Pctl. 75**	0.39	0.19	0.43	0.39	0.19	0.43	0.39	0.19	0.43
**Max.**	0.40	0.19	0.44	0.39	0.20	0.44	0.39	0.20	0.44

Table S2: Biopsy results for the simulations selected by ABC. For all simulated levels of dispersal, the biopsy results closely matched the targets from the published data, supporting the model fit.

Supplemental Figures

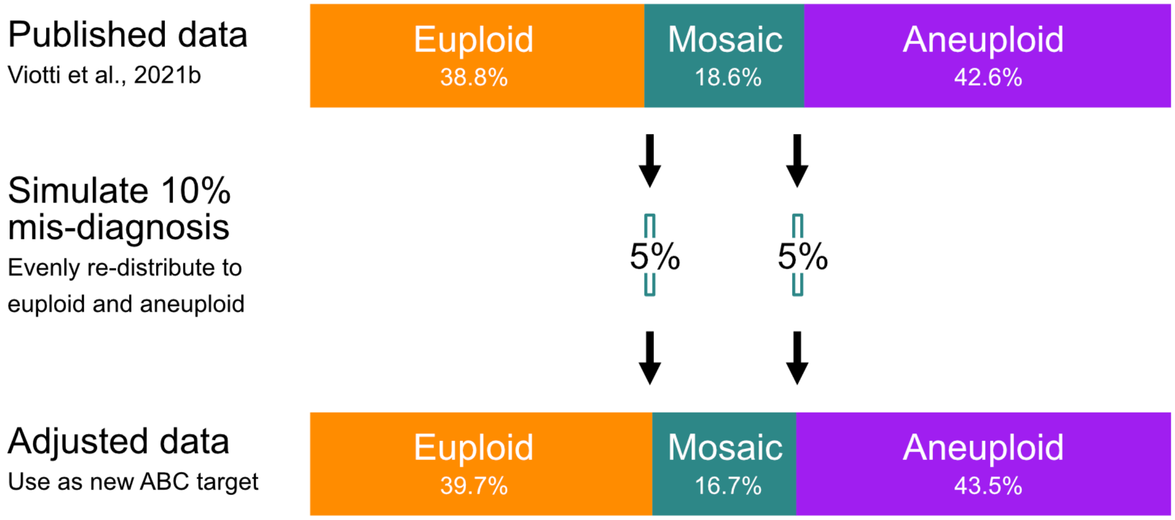

Figure S1: Schematic of approach for simulating misclassification of mosaic embryos. We simulated varying levels of misclassification spanning from 0% to 100% in 10% increments. At each misclassification level, half of the mis-classified mosaic biopsies were assumed to be euploid and the other half aneuploid. These adjusted values were used as the new targets for ABC. The figure depicts an example of simulating a misclassification rate of 10%.
